# Retrieval-Induced Forgetting as Motivated Cognition

**DOI:** 10.3389/fpsyg.2018.02030

**Published:** 2018-10-23

**Authors:** Gennaro Pica, Marina Chernikova, Antonio Pierro, Anna Maria Giannini, Arie W. Kruglanski

**Affiliations:** ^1^ University of Rome, “La Sapienza”, Rome, Italy; ^2^University of Maryland, College Park, College Park, MD, United States

**Keywords:** retrieval-induced forgetting, need for closure, motivated cognition, motivation, memory bias

## Abstract

Recalling information from a particular category can reduce one’s memory capability for related, non-retrieved information. This is known as the retrieval-induced forgetting effect (RIF; Anderson et al., 1994). The present paper reviews studies that show that the RIF effect is motivated. More specifically, we describe research showing that the need for closure (NFC; the motivation to attain epistemic certainty; Kruglanski and Webster, 1996) generally *enhances* the RIF, because this prevents uncertainty and confusion from the intrusion of unwanted memories during selective-retrieval. However, when the content of the to-be-forgotten information serves the retriever’s goals, NFC *reduces* RIF. Overall, the present findings are consistent with the view that motivation can affect the magnitude of RIF effects which, in turn, can serve as a mechanism for reaching preferred conclusions.

## Introduction

A few days ago, the first author was trying to remember a name of a charismatic woman he had met at a party several months ago. After a couple of minutes, he finally managed to remember her name. After that, he also wanted to remember the names of the other people he’d met on that occasion. However, this latter task proved so difficult that he had to give up. It seemed that the name of the charismatic woman kept intruding into his mind whenever he tried to think of the other people’s names, thus making recalling them more difficult.

The episode above is one example of how memory may be affected by the process of selective-retrieval. More specifically, recalling information (e.g., a name of a charismatic woman) from a particular category (e.g., people at a party) can reduce one’s memory capability for related, non-retrieved information (e.g., the names of other party guests). This effect is known as retrieval-induced forgetting (Anderson et al., 1994; Anderson, 2003). Globally, the RIF phenomenon has essentially been explained as an automatic cognitive effect (due to inhibition or interference; see Anderson et al., 1994) induced by partial recall. We propose that the retrieval process (and its effects on forgetting) is not solely cognitive; rather, it is also affected by motivational factors. We further postulate that one motivational factor at heart of the RIF phenomenon is the *need for closure* (NFC; Webster and Kruglanski, 1994). In the current mini-review, we present studies relevant to these suggestions, thus demonstrating that reconstructive memory is governed by the same mechanisms that underlie all motivated judgments (Kunda, 1990; Kruglanski, 1996; Kruglanski et al., 2012).

The RIF effect has been studied using the retrieval-practice paradigm, in which participants memorize a set of category-exemplar pairs (usually eight categories with six items each: e.g., “Fruit-Apple”; “Fruit-Orange”; “Color-White”; Anderson et al., 1994). After the initial study-phase, subjects retrieve half of the items of half of the categories (usually three items of a subset of four categories). Selective retrieval-practice is accomplished using cued stem tests (e.g., “Fruit-Ap___”). After a 20-min distractor task, participants are asked to recall all the exemplars of all the categories originally studied. The *RIF effect* refers to the reduced recall of retrieval non-practiced items (Rp- items; e.g., “Fruit-Orange”) as opposed to non-retrieval practice items from unpracticed categories (Nrp items; e.g., “Color-White”) (RIF = Nrp > Rp-). The RIF is a pervasive phenomenon that occurs in a variety of contexts and with a wide assortment of stimuli, ranging from eyewitness testimony to language acquisition (for a review, see Storm et al., 2015).

According to the present motivational perspective, the RIF effect is a fundamentally adaptive mechanism: it protects the recall of desired and/or useful memories from the interference of undesired and/or useless ones (Storm, 2011). More specifically, the intrusion of interfering items during recall may undermine selective-retrieval, fostering epistemic uncertainty and confusion. People are generally motivated to avoid such confusion during retrieval; as a result, they engage in RIF. One intriguing implication of this analysis is that motivational variables that lead one to have a stronger-than-usual dislike for uncertainty can influence the strength and direction of RIF effects. One such variable is the NFC, described in more detail below.

Need for closure is defined as a “desire for a firm answer to a question, any firm answer, as compared to confusion and/or ambiguity” (Kruglanski, 2004, p. 6). More specifically, NFC is a motivational tendency that leads individuals to seek out clarity, structure, and certainty. As a result of this, individuals who are high on NFC have a strong preference for quick and firm decisions, experience discomfort with ambiguity, and desire stable knowledge that does not change across situations. They are closed-minded and reluctant to accept information that is in conflict with their already-formed opinions. In accordance with this, previous work on NFC has shown that this motivation leads individuals to focus on schema-confirming information and avoid disconfirming information (Dijksterhuis et al., 1996; Macrae and Bodenhausen, 2000; Strojny et al., 2016). It also enhances goal shielding (i.e., when an individual protects his or her focal goal by inhibiting alternative goals; Shah et al., 2002) and increases the efficiency with which individuals handle irrelevant information (Kossowska, 2007).

High NFC individuals are particularly motivated to ignore irrelevant information, because such information can prevent them from attaining clear-cut knowledge. The intrusion of interfering items into the mind during selective retrieval in the RIF paradigm is one case in which irrelevant information (Rp- items) can foster uncertainty as to which focal memories (Rp+ items) should be retrieved at any given moment. Since high NFC individuals have a strong aversion to any type of uncertainty or confusion, they should thus increase their focus on the class of items to be recalled (Rp+ items) while doing their best to forget irrelevant competing items (Rp- items) that could interfere with the retrieval process. In other words, NFC should generally lead to an enhanced RIF effect (Hypothesis 1).

However, when the *content* of the information to be forgotten (Rp- items) is relevant to retrievers’ goals, high NFC individuals should be more likely to keep this information in mind. This occurs because high NFC individuals are motivated to avoid an unpleasant inconsistency between their current desires and the informational content in their mind (Kruglanski and Shteynberg, 2012). Thus, we hypothesize that when the content of the to-be-forgotten information (Rp- items) is manipulated (i.e., to be consistent or inconsistent with retriever’s goals), NFC should enhance the RIF *only* when the to-be-forgotten information does not serve the retriever’s goals. When the to-be-forgotten information serves goals, NFC should *reduce* the RIF effect (Hypothesis 2).

Lastly, work on the role of cognitive resources within goal pursuit has shown that every cognitive process requires a combination of resources and motivation in order to be enacted (Kruglanski et al., 2012). This combination of resources and motivation plays an especially important role when cognitive activity is difficult (vs. easy) to carry out: when a task is easy, anyone can do it, and thus relatively little motivation and/or resources are necessary. However, when a task is difficult, high motivation and/or high resources are required in order to complete it successfully (Kruglanski et al., 2012). Thus, we further expected that Hypothesis 1 should be moderated by participants’ available cognitive resources: individuals with high NFC (i.e., those who are motivated to engage in RIF) should be the most effective at RIF when they also have ample cognitive resources (Hypothesis 3). Evidence for each of these three hypotheses is reviewed in the following section.

## Evidence for RIF as Motivated Cognition

Recent research has demonstrated that the RIF is influenced by NFC, thus providing support for Hypothesis 1. In three studies, Pica et al. (2014) found strong NFC effects on RIF in a Retrieval-Practice Paradigm using eyewitness scenarios. More specifically, in the first two studies, participants read a narrative about a robbery and then viewed ten characteristics (e.g., age, build, haircut, and face shape) ascribed to each of the two protagonists of the robbery (one blond and one dark-haired), for a total of 20 items. To induce the RIF effect, half of the participants practiced with half of the blond-haired offender’s characteristics and the second half practiced with half of the dark-haired offender’s characteristics. The results showed that high NFC participants displayed a higher RIF effect, both when assessing memory with a free-recall-task (Study 1) or with a more controlled memory task (i.e., using the first two letters to control for output interference; Study 2). Study 3 replicated this effect while varying the witness scenario: participants read two narratives about two burglaries in two houses where ten items (e.g., a television) were stolen from each house. The results of this study again showed that high NFC participants exhibited a higher RIF effect (Hypothesis 1).

Although the foregoing studies showed that the RIF effect is influenced by motivational variables, and by the NFC in particular, they did not show that motivation can be sensitive to the *content* of what is forgotten. Consistent with our Hypothesis 2, other research has found that motivation can reduce or enhance the RIF effect to the extent that the Rp- items serve the retriever’s goals (or do not serve them). For instance, Pica et al. (2016) found that self-threat, a condition known to enhance prejudicial responses (Fein and Spencer, 1997), biased individuals’ memory of homosexual targets. In particular, participants under self-threat (which was induced by fake negative feedback on an IQ test) enhanced the RIF of positive items ascribed to a homosexual target, and reduced the RIF of negative items ascribed to the same person. Standard RIF effects were observed for both positive and negative stimuli ascribed to a heterosexual target. Importantly, the results in the self-threat condition were conceptually replicated with another stigmatized target, i.e., African American target (see Pica et al., 2017).

These results suggest that when people are motivated to arrive at a particular conclusion, they tend to construct a coherent justification for that conclusion by (1) avoiding disconfirming information; and (2) activating information that is consistent with their goals and that is useful for reaching desired conclusions (Santioso et al., 1990; Kruglanski, 1996; Bélanger et al., 2014).

According to our Hypothesis 2, the effect described above should be strengthened by individuals’ NFC. In line with this reasoning, recent research has shown that high NFC individuals are affected by gender-role-stereotypes when remembering characteristics of female managers (Pica et al., 2018). In two studies, high NFC individuals enhanced the RIF effect of *masculine* dimensions (e.g., *powerful* and *agency*) ascribed to a female manager and reduced the RIF effect of *feminine* dimensions (e.g., *warmth* and *communal*) ascribed to the same target. Furthermore, as expected, the recall of *feminine* dimensions as ascribed to the female manager were less impaired by high NFC participants, as compared to the recall performance of the same dimensions when ascribed to the male manager; and the recall of *masculine* dimensions ascribed to the female manager were more strongly impaired than when ascribed to the male manager. The present findings suggest that when faced with women leaders, high NFC individuals are motivated to search for gender stereotype-congruent memories, thus reducing the RIF of such memories. More broadly, these results indicate that NFC focuses the attention of the retriever on memories that are consistent with their prior stereotypical knowledge, by enhancing the association between stereotypical memories (*communal* and *feminine* dimensions for the female manager and *agency* and *masculine* dimensions for the male manager) and the target, and reducing the association between counter-stereotypical memories and the same target.

Finally, research has also shown that the relationship between NFC and RIF is moderated by energy availability (Hypothesis 3). In two experiments, Pica et al. (2013a) found that when forgetting interfering items was difficult (i.e., participants had only a small amount of practice with the focal items), and participants had adequate cognitive resources, RIF was positively affected by participants’ NFC. In Study 1, participants’ NFC and circadian rhythm was measured. One week later, they came back to the lab to perform a memory experiment either at times that matched their circadian rhythm (e.g., morning people in the morning) or at times that did not (e.g., morning people in the evening). During this second session, participants completed the RIF paradigm utilizing neutral stimuli. In this experiment, all participants were placed in a condition where forgetting interfering items was difficult (participants had a small amount of practice with the focal items). The results revealed that high NFC participants with high energy (e.g., those who came in at times of testing that matched their circadian rhythm) exhibited a greater RIF effect. In Study 2, the authors added a condition where forgetting interfering items was easy (i.e., participants had a large amount of practice with the focal items). Participants’ scores on the Automated Operation Span Task (OSPAN; Conway et al., 2005) were used as a measure of resource availability. Then, participants completed the RIF paradigm using brand names of product categories. In line with the hypotheses, the findings of Study 2 confirmed that when the amount of retrieval-practice was high (easy condition) no differences in the RIF were found; however, when the amount of retrieval-practice was low (difficult condition), high NFC participants with a high OSPAN score were those with the highest RIF effect. These results indicate that, as predicted, the NFC is most likely to enhance RIF when individuals have adequate cognitive resources.

## Toward a Motivational View of the RIF: Re-Interpreting Prior Findings

As can be seen above, multiple studies have provided direct support for our notion that the RIF effect is subject to motivational influence. In addition, our motivational viewpoint is broadly compatible with previous research on RIF: even prior studies that did not directly examine the role of motivation in RIF can be interpreted as fitting the motivational perspective. In this section, we describe such previous findings and explain how they can be viewed through a motivational lens.

Garcia-Bajos and Migueles (2009) demonstrated that stereotype activation prevented RIF for highly stereotypical traits (e.g., Athlete–Competitive, when participants selectively practiced low-typicality traits). On the contrary, RIF was normally found for low-typicality (Athlete–Risky) or control traits (i.e., a stereotyped trait associated to a person’s name; e.g., Mikel–Competitive). Consistent with the above findings, Dunn and Spellman (2003) found that stereotypical belief (i.e., the extent to which people believe in the stereotype) impaired the later recall of (i.e., RIF for) *stereotypical* traits ascribed to a target [e.g., June (Asian-Americans)-Studious], when *individuating* traits (ascribed to the same target) were practiced [e.g., June (Asian-Americans)-Elegant]. The foregoing findings have previously been explained in terms of the cognitive mechanism of *integration* between the items. Specifically, the argument was that the stereotype promotes the creation of strong linkages among the stereotypical traits, which then circumvent the RIF effect. However, these same findings can be explained as an effect of *motivation*: individuals are motivated to uphold their stereotypical beliefs, thus reducing the RIF for information consistent with those beliefs.

Moreover, Storm and Jobe (2012) found a positive relationship between levels of RIF and a tendency to recall significantly fewer negative events on an autobiographical memory task. In explaining these findings, the authors argued that people with a greater inhibitory ability are more likely to inhibit (enhanced RIF) negative autobiographical memories. However, a *motivational* bias toward remembering positive features about the self, thus maintaining one’s positive self-image and positive affect, could also cause individuals to forget negative events. Consistently, a recent study (Nuney et al., 2017) demonstrated that people high in state anxiety show the highest RIF for threatening categories, thereby protecting the self.

Furthermore, Coman and Hirst (2015) found that socially shared RIF (i.e., RIF induced by a speaker to a listener) is higher when the speaker and the listener belong to the same group (ingroup) rather than a different group (outgroup), thereby showing that this effect is contingent to a motivational tendency to promote the emergence of shared mnemonic representations that preserve group identity.

Amir et al. (2001) found that people with generalized social phobia did not show RIF for negative social words. Similarly, Kuhbandner et al. (2009) showed that the RIF of negative stimuli decreased as a function of the participants’ dispositional negative affectivity. The authors proposed item-specific processing, which is known to eliminate the RIF, as an explanation of this effect. However, an alternative motivational explanation is also possible: dispositional negative affectivity may drive participants toward a mood-congruency bias (i.e., the *motivation* to remember items congruent with the participants’ affective state), thus reducing the RIF of negative items.

Thus, in our view, all of the findings described above fit well with the notion that the RIF effect is motivated. This suggests that, in line with our hypotheses, motivation is a critical but heretofore overlooked determinant of the RIF effect.

## Discussion

The present work aims to contribute to the RIF literature by elucidating the motivational dynamics that underlie the phenomenon. We hypothesized and found that NFC plays a central role in determining the extent of RIF effects. Specifically, NFC was expected to lead to greater RIF in order to avoid the confusion and uncertainty that may stem from the interference of unwanted items during selective-recall (Hypothesis 1). However, where the *content* of items to be forgotten was manipulated, NFC was expected to lead to higher RIF effect only when the to-be-forgotten-items do not serve the retriever’s goals. When the to-be-forgotten-items serve the retriever’s goals, then the RIF of such memories is reduced (Hypothesis 2). Moreover, individuals with high NFC should show the greatest RIF effects when they also have high cognitive resources (Hypothesis 3). We reviewed data that provided support for all three of these hypotheses.

One implication of the above reasoning is that over time, selective-retrieval may serve to affirm and re-affirm previous knowledge and desired conclusions. This can have important consequences for intellectual rigidity and arrogance. In fact, RIF may serve to confirm prior epistemic hypotheses by enhancing the accessibility of consistent information and reducing the accessibility of inconsistent information in memory, therefore reducing the possibility of hypothesis-invalidation. Pessimistically, this view of the RIF seems to leave no space for changing one’s prior ideas or invalidating prior epistemic hypotheses. However, conditions in which RIF may lead to forget goal-congruent (hypotheses-validating) items must then be present. A possible moderating factor may be the importance of focal goals: when it is of low importance a similar RIF effect with both consistent and inconsistent information is expected. Furthermore, given that previous work has clearly shown that the RIF is energy-dependent (e.g., Pica et al., 2013b), and consistent with the cognitive energetics theory (CET, Kruglanski et al., 2012), conditions that determine energy depletion should moderate goal-congruency effects of RIF. For instance, the goal-congruency effects we described earlier (Hypothesis 2) should result in greater RIF especially when participants also have energy (or the task is easy). These matters could be profitably explored in future research.

Another avenue for future research may be to investigate the boundary conditions of the effects of motivation on the cognitive processes that underlie RIF. One hypothesis is that when memories are neutral with regards to the (specific) motivational and emotional states of the individual (i.e., directed to confirm prior epistemic hypotheses), RIF effects are determined by cognitive processes only (e.g., inhibition and interference), and/or by an interaction between cognitive processes and non-specific motivations such as non-specific NFC (i.e., non-directed to confirm prior epistemic hypotheses).

In line with this latter idea are two recent papers showing that non-specific motivations (i.e., rewards, mastery approach goals, and performance approach goals) influence the cognitive processes underlying the RIF on neutral stimuli. More specifically, rewards (i.e., apple juice during retrieval-practice), and mastery approach goals (i.e., developing one’s own skills) eliminate RIF, while performance approach goals (i.e., performing better than others) promote RIF (Imai et al., 2014; Ikeda et al., 2015). The lack of RIF under (1) reward and (2) mastery approach goals is due to the fact that these motivations promote connections (and thus integration and spreading of activation) among the items within a category. In this way, competition is reduced, and thus the *inhibition* of the Rp- materials is unnecessary for protecting the selective retrieval of Rp+ items. Yet, it is possible that when competition between neutral memories is too strong to be reduced, or the connections between them are too durable, the effects of motivation disappear (if the motivational force is not enough strong to overcome them); in these cases, the cognitive mechanisms are the only cause of RIF. Future research may shed light on this hypothesis. All in all, however, we believe that cognitive mechanisms generally interact with and/or respond to motivational (conscious and unconscious) forces whenever the person finds the opportunity and elements that serve attainment of desired (specific and non-specific) conclusions.

In conclusion, the role of motivation in memory retrieval effects appears to have important implications for the real social world. For instance, as we have seen so far, motivation can distort memories, and thus possibly also the subsequent evaluations of other persons, brands, and events from the past. In line with this idea, in our studies we investigated the effect of NFC on RIF in various domains (e.g., brand memory, eyewitness memory, and social cognition). Future studies should explore the generalizability of our hypotheses in other contexts where RIF has been proven to have implications (e.g., autobiographical memory, learning, education, and creative thinking; for a review of the applicability of RIF, see Storm et al., 2015). Therefore, during interrogations of eyewitnesses, in education at school, or simply when remembering one’s past experience or another person’s features, people who remember or professionals who induce retrieval must be aware that motivation may have detrimental effects on memory.

## Author Contributions

GP and AP contributed to conception of the study. GP, MC, and AP wrote the first draft of the manuscript. All authors contributed to manuscript revision and read and approved the submitted version. All authors contributed to the first draft of the paper.

## Conflict of Interest Statement

The authors declare that the research was conducted in the absence of any commercial or financial relationships that could be construed as a potential conflict of interest.
